# Accurate Electroadhesion Force Measurements of Electrostrictive Polymers: The Case of High Performance Plasticized Terpolymers

**DOI:** 10.3390/polym14010024

**Published:** 2021-12-22

**Authors:** Amaury Fimbel, Thierry Abensur, Minh-Quyen Le, Jean-Fabien Capsal, Pierre-Jean Cottinet

**Affiliations:** 1 Electrical Department, Ladoua Campus, University Lyon, INSA-Lyon, LGEF, EA682, 69621 Villeurbanne, France; amaury.fimbel@insa-lyon.fr (A.F.); minh-quyen.le@insa-lyon.fr (M.-Q.L.); jean-fabien.capsal@insa-lyon.fr (J.-F.C.); 2 ArianeGroup SAS, 66 Route de Verneuil, 78130 Les Mureaux, France; thierry.abensur@ariane.group

**Keywords:** electroadhesion, Maxwell pressure, electrostrictive polymer, material optimization

## Abstract

Electroadhesion is a phenomenon ruled by many characteristic intrinsic parameters. To achieve a good adhesion, efficient and durable, a particular attention must be provided to the adhesion forces between the involved parts. In addition to the size and geometry of electrodes, parameters of materials such as dielectric constant, breakdown electric field, and Young’s modulus are key factors in the evaluation of electroadhesion efficiency for electrostrictive polymers and electroactive devices. By analyzing these material parameters, a method is proposed to justify the choice of polymer matrices that are fit to specific electroadhesion applications. Another purpose of this work aims to demonstrate a possibility of accurately measuring the electroadhesion force. This physical parameter has been usually estimated through equations instead, because of the complexity in setup implementation to achieve highly precise measure. Comparisons based on the parameters criterion reveal that besides the intrinsic properties of material, some other parameters relating to its physical phenomena (e.g., saturation of dipolar orientation under high electric field leads to decrease dielectric constant), or physical behavior of the system (i.e., surface roughness reduces the active electrode area) must be thoroughly considered. Experimental results pointed out that plasticized terpolymer leads boosted electroadhesion performance compared to the other counterparts, up to 100 times higher than conventional polymers. The developed materials show high potential in applications of active displacement control for electrostrictive actuation.

## 1. Introduction

Electroadhesion was discovered by two Danish scientists, Alfred Johnsen and Knud Rahbek, at the beginning of the 20th century [1]. It was firstly described as the electrostatic attraction between two materials when an electric field is applied between them [2]. Dedicated to communication at its early stages of development, it is now mainly used for soft robotic applications [3,4,5,6] thanks to its easy force control via applied voltage, and the capability to pick up fragile objects. Even if performances of different materials have been explored such as composite and elastomer [7,8,9,10,11], the principal materials used to design such electrostatic actuators are electrostrictive polymers, because they are light-weight, affordable, flexible, controllable to hold atypical shapes, and easily customized for different applications versus traditional grippers materials [12,13,14]. These polymers, despite their important strains and decent forces, need a high voltage excitation to reach sufficient mechanical deformation. Among them, a relaxor poly(vinylidene fluoride-trifluoroethylene-1,1-chlorofluoroethylene/chlorotrifluoroethylene) terpolymer (abbreviated as P(VDF-TrFE-CFE/CTFE)), when being doped with a plasticizer, shows an excellent electromechanical response [15]. Commonly used as actuators [16,17,18] or sensors [19,20,21,22,23,24], the terpolymer also exhibit particularly impressive abilities when it comes to electroadhesion. For instance, it has been demonstrated that fluorinated terpolymer can hold a frictional shear stress of 21 N/cm^2^ when subjected to an electrical field of 24 V/µm [25]. To be able to apply such an important electric field, experiments were conducted on very thin films (i.e., a few tens of micrometers), which were fragile to perform any actuation. As a matter of fact, the sample was deposited on a metallic woven textile substrate, making it possible to reinforce the structure as well as to ensure electroadhesion performances.

**Figure 1:**
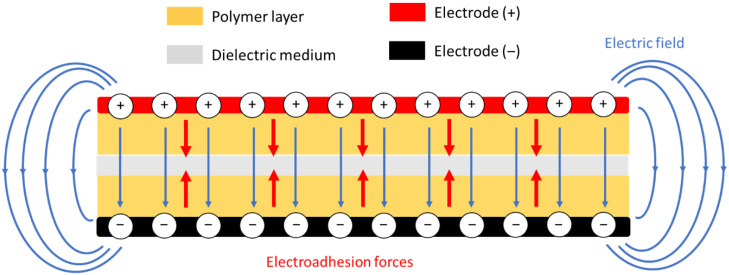
Mechanisms of Electroadhesion.

In this study, we merely focus on the phenomenon at low electric field and without the influence of a substrate. The main purpose here involves analyzing the electrostatic interaction of a simple architecture consisting of two electrostrictive polymers separated by a dielectric medium (e.g., another polymer, liquid, air, vacuum, etc.) and sandwiched between two electrode layers. Figure 1 shows the mechanism of interactions leading to electroadhesion. When being surrounded by an electric field, electrodes tend to charge either positively or negatively. A positive electrode (in red) creates an attraction to a negative one (in black) and vice versa, leading to the compression of the electroactive polymer (in orange) and to the reduction of thickness of the air or vacuum layer in between (in grey). After actuation, charges in the electrodes can also be useful for electrostatic attraction with surrounding objects. Adhesion can be led by both mechanical and electrostatic aspects. The dielectric constant of the material helps to enhance this attraction that can lead to grip, actuation, or adhesion. Research is currently focusing on improving these electroactive capabilities, mainly in a composite elaboration way, by doping electroactive properties of polymer matrices with fillers, for example [26]. 

Using the above arguments, this paper reports on the main characteristics of materials that can have an impact on the performances of electroadhesion. To enhance the electroadhesion, we propose here a novel approach relying on intrinsic dielectric parameters of electrostrictive polymers. The main challenge is to achieve the electrostatic force measurement, which considered to be a key factor reflecting the adhesion quality. To date, methods used for empirically performing force measurement are still rarely investigated in the literature. This work therefore suggests a new method to achieve the experimental value of the interaction force, which is counterbalanced by weights under the equilibrium regime. Finally, different relevant parameters of the developed materials dedicated to the actuation ability are then compared between the empirical data and theoretical model. After a brief presentation of opportunities and challenges, benchmark and results are analyzed and discussed.

## 2. Method for Achieving High Performances of Electroadhesion

### 2.1. Equations Governing Electroadhesion Forces

Electroadhesion is the force generated from the electrostatic field and acts between the two surfaces. Usually, the normal attractive force of electrostrictive material is known as [27]:

(1) $$F_{N}=\frac{\epsilon_{r}\epsilon_{0}A}{2}E^{2}=\frac{\epsilon_{r}\epsilon_{0}A}{2}\frac{V^{2}}{d^{2}}$$

where $F_{N}$ is the normal force of attraction in a static contact; $\epsilon_{r}$ is the relative permittivity of the material; $\epsilon_{0}$ is the vacuum permittivity ($i.e.,\;\;\epsilon_{0}=8.85\times 10^{-12}\;F\cdot m^{-1})$; A is the overlap area of the electrodes, E and *V*, respectively, denote the electric field and the applied voltage; d denotes the distance between the two electrodes.

According to the model of Equation (1), there is a quadratic dependence of the force with respect to the electrical field, meaning that the electroadhesion of material can be substantially improved by applying sufficiently high input voltage. However, this value is usually limited by the breakdown electrical field ($E_{Bd}$), above which the electrostrictive material collapses on itself and drastically deteriorates. For a given applied electric field, it is possible to enhance the attractive force by using an electrostrictive polymer with high dielectric strength, i.e., represented by the intrinsic parameter of material ($\epsilon_{r}$).

In practical applications, it is also relevant to use the friction force ($F_{F}$) tangential to the samples [25], which can be expressed as:

(2) $$F_{F}=\mu \frac{\epsilon_{r}\epsilon_{0}A}{2}E^{2}=\mu F_{N}$$

where *µ* is the static friction coefficient between two materials, which depends on interactions between materials and surface roughness. 

To the best of our knowledge, there is no fully reliable analytical model dedicated to the static friction, whose value is in large interval and generally determined via empirical method. As a matter of fact, many parameters such as roughness, asperities, Van Der Waals interactions, mechanical characteristics of material, contact condition, elasticity of tribosystem, lubricant film properties, time of contact, etc., might substantially affect the static friction [28,29,30,31]. Among them, contact between surfaces plays an important role. Most surfaces are rough at the microscale and thus, the real area of contact is only a fraction of the nominal area. Interactions between surfaces like the normal and shear forces happen across this small real area of contact [32]. Contact area is determined by several factors: surface topography, material properties, the applied load, sliding speed, etc. Since the applied load is sustained over a small area, stresses at the contacts can be high and time-dependent properties of the material become important. The macroscopic friction resulting from the collective and interactive behavior of a population of microscopic contacts shows complex time and history dependence [33].

From an experimental point of view, force is one of the physical parameters that can be easily real-time monitored to assess the electroadhesion performance. Instead of investigating on the prediction of the friction coefficient, we develop in this study a novel experimental setup enabling to identify the electroadhesion force. Thus, for an electrode surface standardization (i.e., denoted *A*), the following Maxwell pressure ($P_{M}$) can be deduced from the force measurement:

(3) $$P_{M}=\frac{\epsilon_{r}\epsilon_{0}}{2}E^{2}=\frac{F_{N}}{A}$$

As can be seen, $P_{M}$ is linearly proportional to the dielectric constant $\left(\epsilon_{r}\right)$, which is considered as a key parameter having strong impact on the resulting electrostatic force. The above equation demonstrates a further advantage of increasing the permittivity in order to achieve a better electroadhesion. According to Equation (2), it is possible to deduce the pressure generated from the friction force ($F_{F}$) in the case of a sliding tangential motion:

(4) $$P_{F}=\mu \frac{\epsilon_{r}\epsilon_{0}}{2}E^{2}\;\;\;\propto \;\mu \epsilon_{r}$$

This pressure is proportional to the dielectric constant and the static friction coefficient (*µ*) of the material, simultaneously. For a sake of simplicity, our study here merely focuses on the normal contact between two polymer surfaces. Sliding tangential motion is a complex situation, and thus will not be specifically detailed in this investigation. Furthermore, an optimal electroadhesion phenomenon does not only rely on its excellent dielectric constant, but also on other intrinsic parameters that strongly affect the ability of actuation. In the following, two relevant parameters, electrical breakdown and mechanical breakdown, are introduced to better clarify this issue.

### 2.2. Electrical Breakdown

Electrical breakdown is a process that occurs when the electric field caused by applied voltage exceeds the dielectric strength of the material [34]. Each material has its own range of electric field for operation that affects its performances and life duration [35]. A more convincing explanation can be given here with the use of three different electrostrictive materials: a pure polymer and two modified polymers (A and B) with the same matrix but doped with plasticizers. The percentage of plasticizer is higher in A than in B. 

**Figure 2:**
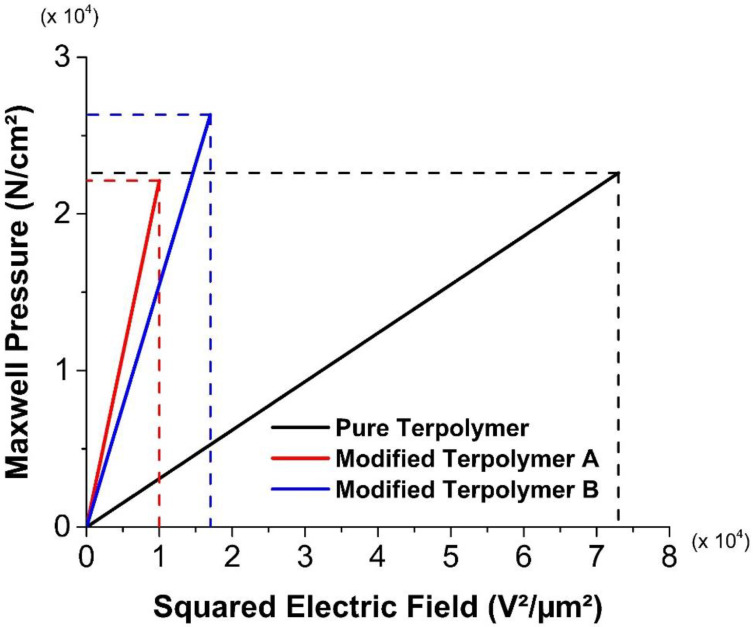
Maxwell pressure versus squared electric field for different electrostrictive materials. Focus on electric breakdown.

According to Della Schiava et al. [36,37], the dielectric constant of our electrostrictive material significantly improves with the increasing plasticizer content. Hence, the incorporation of plasticizer in polymer matrix results in enhancement of electroadhesion performances. Figure 2 highlights that for the three materials, the Maxwell pressure grows linearly with the squared electrical field and the corresponding slope equal to $\frac{1}{2}\epsilon_{r}\epsilon_{0}$, according to Equation (2). As expected, polymer A has the highest slope value of around 2.2 × 10^8^ N/V², and so exhibits a better Maxwell pressure at a given electric field. For instance, under an excitation of 100 V/µm, sample A can result in a pressure of 220 MPa, while 160 MPa and only 35 MPa have been recorded in the case of the sample B and the pure terpolymer. Nonetheless, the electrical breakdown field of A (~100 V/µm) is lower than the others, which can lead to reduced electroadhesive performance [38]. The pure polymer, on the other hand, exhibits a larger range of electrical breakdown (~270 V/µm) but shows a low value of slope, 0.3 × 10^8^ N/V² approximately, which is eightfold less than the one of the polymer A. Sample B seems to be a good compromise between the electrical breakdown limit and the electroadhesion performance, allowing to achieve a higher limit of Maxell pressure (~2.6 MPa) with respect to the others (~2.25 MPa). Despite the saturated electric field of the interfacial phenomena being much lower than that of the pure terpolymer, the modified terpolymers (samples A and B) still lead to far superior performance in terms of electroadhesion response. Accordingly, the application and the environment of use will decide the choice of the suitable material and a fair balance must be ensured to fulfill the requirements: High adhesion or low electric consumption.

### 2.3. Mechanical Breakdown

The second parameter that affects the electroadhesion phenomenon is the mechanical breakdown. Electrostrictive materials have a wide variety of mechanical behavior; some can bear important stress [25] while others can deteriorate more easily. Under important solicitations, mechanical breakdown can occur, provoking cracks and greatly reduced electroadhesion performance, even nullified in some cases. 

**Figure 3:**
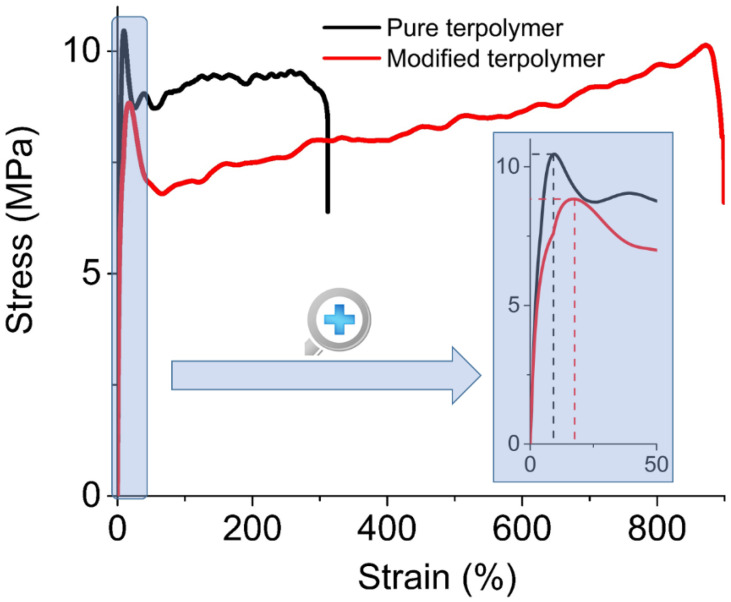
Stress-strain characteristics of the pure and modified terpolymers. Inset on the right-hand side is a focus on low strain.

Another explanation of this breakdown is illustrated in Figure 3. As observed, terpolymer incorporated with plasticized gives rise to a higher critical strain level, which agrees with the fact that Young’s modulus of the pure terpolymer is superior to the modified one [39]. Actually, the plasticizing effect enables to considerably improve the influence of the defect in the polymer chains, leading to a strain of three times greater in the case of the modified terpolymer with respect to the pure one. The introduction of the plasticizer into the polymer matrix increases the molecular mobility and consequently decreases the Young modulus of the fluorinated terpolymers, confirming why under similar applied stress, the plasticized sample results in higher deformation. In other words, the modified terpolymer is much more flexible, explaining why it exhibits higher strain range, so higher mechanical breakdown limit.

Concerning the stress of the materials, the inset shown in Figure 3 zooms in on the linear elastic behavior of both samples, where the deformation does not excess 20%. Logically, the pure sample leads to higher peak in the stress compared to the modified one, which agrees to its higher Young modulus. Interestingly, the peaks of the two samples are not proportional to their Young modulus (respectively, around 100 MPa and 45 MPa), as they do not occur at the same strain. Concretely, the pure sample has a maximum stress of 10.4 MPa at 10% deformation, while 8.8 MPa at higher strain (19%) for the plasticized sample. Consequently, it can be appeared that the two maximum stresses are not significantly different as they should be expected.

Evolution of the stress beyond 100% of strain presents an interesting behavior. From 100% to 300%, both materials exhibit so-called strain hardening characteristics [40]. After the yield point (at respectively 10% and 19% for the pure and the modified terpolymer), samples turn out to be softening due to their mechanical degradation, which is manifested by an abrupt decrease in the stress. Subsequently, a saturation regime quickly appears where the stress becomes constant. The chain molecules tend to orient and align in the direction of the mechanical solicitation, resulting in an increase in the material’s stiffness in that direction. Such a hardening behavior is more visible for polymers with higher molecular mobility, which corresponds to the plasticized terpolymer. This explains why the stress of the pure material seems to be unchanged whereas the one of the plasticized material somewhat increases as a function of the stress. Experimental data show that in a range of 300% of deformation, the pure and the modified terpolymer lead to a moderate increase of 10% and 15% in the stress, respectively. Regarding the modified sample, its stress increases more significantly at above 500%. This range is obviously out of the mechanical strength limit of the pure terpolymer whose breakdown occurs near to 300%.

Considering all the above analyses, it can be concluded that the mechanical breakdown is one of the key parameters that has a strong impact on the electroadhesion efficiency. Accordingly, materials should be used in a specific range of strain, preferably below the yield point to avoid any mechanical degradation. 

Finally, the following subsection propose a method to compare the performances of several electrostrictive polymers. Criteria are based on the dielectric constant as well as the allowable maximal voltage and/or mechanical solicitation imposed by the material.

### 2.4. Selection of Polymer Matrix

This section aims to justify the choice of electrostrictive polymers suitable for application in electroadhesion, based on previous observations and criteria. Four materials are investigated, including (1) Polyethylene Terephthalate (PET); (2) pure Fluorinated Terpolymer P(VDF-TrFE-CFE); (3) P(VDF-TrFE-CFE) doped with 10wt%. of Diisononyl Phthalate (DINP) plasticizer (called modified terpolymer); and (4) Polyurethane Shore 87 (PU87). Table 1 summarizes the relevant parameters of these polymers, which consist of the dielectric permittivity ($\epsilon_{r}$), the friction static coefficient (*µ*), the Young modulus (Y), the breakdown electric field, and the product μϵ Pressure of shear stress ($P_{F}$ according to Equation (4)).

**Figure 4:**
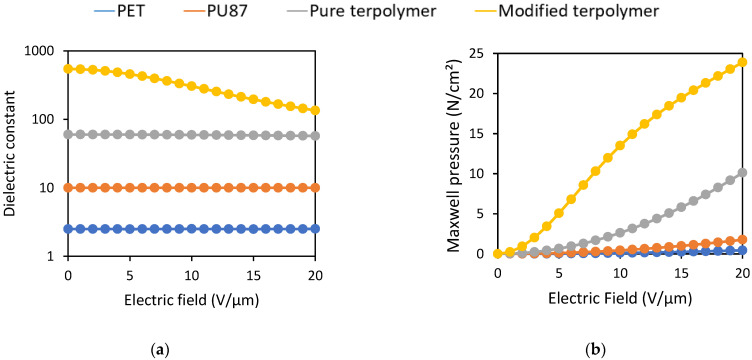
Representation of material performances for electroadhesion: (**a**) Dielectric Constant (**b**) Maxwell Pressure.

Figure 4a illustrates the measured dielectric constant as a function of the electric field for all materials described above. It can be noticed that both pure and plasticized terpolymers have a significant dielectric permittivity under very moderate electric field. The modified terpolymer exhibits 5-fold increase of dielectric constant with respect to the pure one. Obviously, the plasticized terpolymer allows one to boost the electroadhesion, but under higher voltage applications, variation of the relative permittivity should be considered because of the saturation of the dipole’s polarization. This saturation can be predicted thanks to the Debye–Langevin formalism [43]. To reduce any unexpected effects originated from high electric field, this study therefore focuses only on a low electric field that is limited by 5 V/µm. Compared to all the other materials, the PET shows a very low dielectric constant. The PU87 has a low dielectric constant too, but its high static friction coefficient (*µ*) makes it possible to attain significant friction force (Equation (2)). Figure 4b illustrates the expected behavior of the Maxwell pressure versus electric field for all electrostrictive materials. Estimation of this pressure is relied on Equation (3), where the dielectric constant of Figure 4a is used. The plasticized terpolymer is revealed to be the most appropriated candidate for enhancing electroadhesion, which is indicated by the dielectric constant as well as the Maxwell pressure. The pure terpolymer, due to its significant breakdown strength and high stability in dielectric permittivity for a large range of electric field, seems to be somehow more interesting in higher voltage application. Accordingly, the results clearly demonstrate excellent benefit of the terpolymer for electrostatic actuation (whether the plasticizer is included or not) as opposed to the conventional electroactive polymer like PET and PU87.

## 3. Fabrication Process and Characterization Test Bench

### 3.1. Sample Preparation

**Figure 5:**
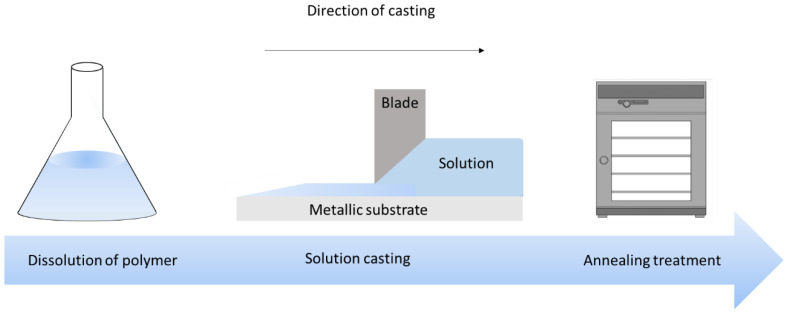
Sample preparation.

All samples are elaborated through the solution casting method to perform thin films with thickness of around 100 to 120 µm, and rectangular shape of 60 mm × 30 mm dimensions. The fabrication process is briefly illustrated in Figure 5.

PET films (Mylar RS 785-0792, Radiospares, Beauvais Oise, France) are cut from a manufactured A4 sheet size. Fluorinated Terpolymer samples are made from P (VDF-TrFE-CFE) terpolymer powder provided by Piezotech S.A.S. (Arkema group, Lyon, France). First of all, the powder is dissolved in Methyl Ethyl Ketone solvent (MEK, Sigma-Aldrich, Paris, France) with proportions of 14 wt.% powder and 86 wt.% MEK. After magnetic stirring, the solution is left at room temperature for at least 2 h. To prepare the modified Terpolymer, Diisononyl Phthalate (DINP, Sigma-Aldrich, Paris, France) plasticizer is added to the matrix and stirred again for 18 h. Then, the solution is cast with a Doctor Blade (Elcometer, Manchester, UK), and then left at room temperature during 3h for completely evaporating the MEK solvent. To increase the crystallinity of the samples, annealing at 98 °C is performed during 2 h in an oven (UFE 400, Memmert, Schwabach, Germany).

The PU 87 (Estane 58887 NAT 038, Sigma-Aldrich, Paris, France) is made of a co-block polymer composed of 4,4-methylene diphenyl diisocyanate (MDI) and 1,4-butanediol (BDO) as hard segments, together with Poly Tetra Methylene Oxide (PTMO) as a soft segment. These materials were purchased from Sigma-Aldrich, Paris, France. Hardness of the sample is around 87 Shore A according to datasheet. PU 87 granules are dissolved in *N*,*N*-dimethylformamide (DMF, Honeywell D158550, ≥ 99.9%) with weight ratio of respectively 20–80% under 80 °C during 3 h with mechanical stirring. The mixed solution is then left at room temperature for 24 h to degas. After that, the solution is cast on the glass plates, similarly to the other materials as described above. Films are heated at 60 °C for 24 h in the oven, removed from the plates and heated again at 125 °C during 3 h for annealing treatment.

Lastly, all four samples are gold-coated on a single side with thickness of approximately 24 nm using a Cressington Sputter Coater (208HR, Orlando, FL, USA). Two shapes of electrodes are designed: a circular one with 16 mm diameter, and a rectangle one with dimensions of (55 × 20) mm^2^.

### 3.2. Experimental Setup of the Electroadhesion Test

**Figure 6:**
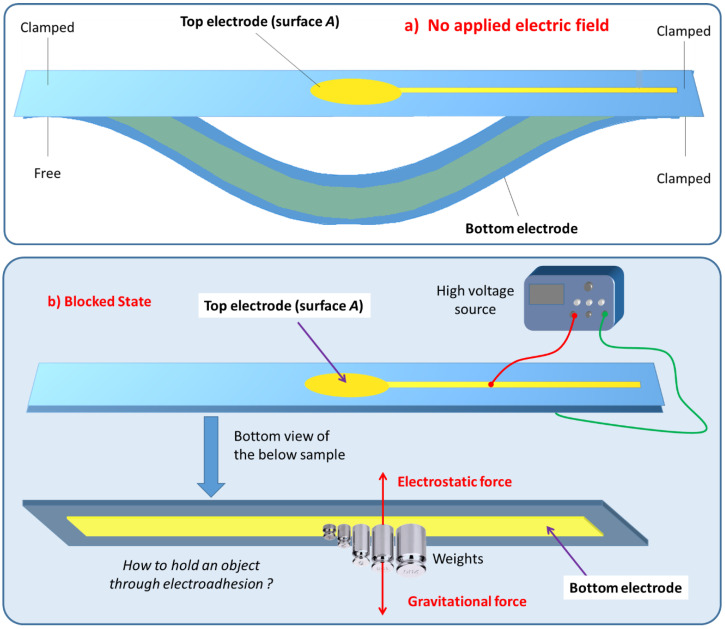

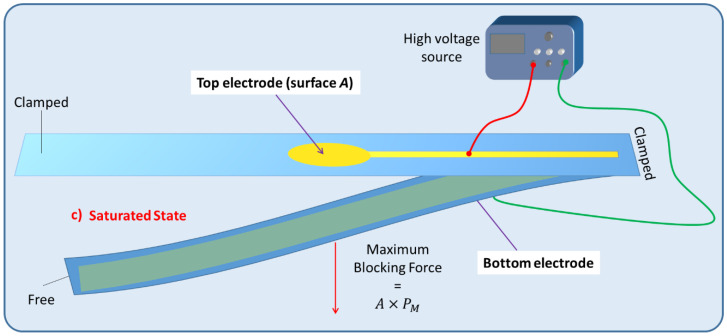
Experimental Bench for electroadhesion measurements. (**a**) Without electric field. (**b**) With electric field and in blocked state. (**c**) With electric field and in saturated state.

Figure 6a illustrates the electroadhesion test of Maxwell pressure generated by electroactive materials described above. Two samples of thin films are facing each other. One is coated with a circular electrode on the top whereas the other is coated with a rectangle electrode underneath (defined as ground). The above sample is clamped on both sides while the bottom one is clamped only on one side and free on the other one. In order to reduce deformation and risks of mechanical breakdown, all soft samples are bound to passive PET layer with adhesive tape (3M ATG 969, 100 µm, 3M, Cergy-Pontoise, France).

**Figure 7:**
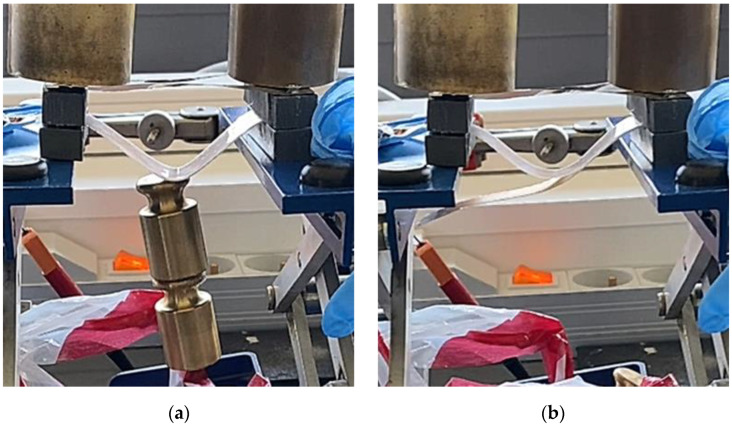
Pictures of the experimental setup where the input voltage is (**a**) ON; and (**b**) OFF.

To achieve electroadhesion, a direct current (DC) high voltage, driven from a function generator (Keysight Technologies, Agilent 33220 A, Santa Rosa, CA, USA) coupled by a high voltage amplifier (Trek 609D-6, Trek Inc, Lockport, NY, USA), is applied between the circular electrode and the ground. Direct measure of the electroadhesive force via a load cell is a real challenge because of its complex implementation to achieve highly accurate measurement. As a result, our idea here is to estimate the normal static force through calibrated weights exerted on the bottom sample (Figure 6b). When the high voltage is ON, the electroadhesion occurs and a force sticks the samples together, the weight applied to the bottom is counterbalanced by electrostatic force. For a given voltage, experiments are repeated with an increasing value of weight. The maximum force before release, corresponding to a transition between the blocked and the saturated state (Figure 6c), is defined as the empirical normal force $F_{N}$. Based on this measure, it is therefore possible to deduce the Maxwell pressure given by Equation (3), dividing the empirical normal force $F_{N}$ by the electrode area A. To better show the experimental test, videos is available in the supplementary materials (Video S1: Holding a 200g weight using an applied field of 5.5 V/µm; Video S2: Lifting a 10g weight using an applied field of 15 V/µm). Figure 7a displays the experimental benchmark of a modified terpolymer holding a 200 g weight under an applied electric field of 5.5 V/µm (800 V). As soon as the high input voltage is OFF, the below sample immediately releases (Figure 7b), confirming the quick response of electroadhesion actuation. 

## 4. Results and Discussions

**Figure 8:**
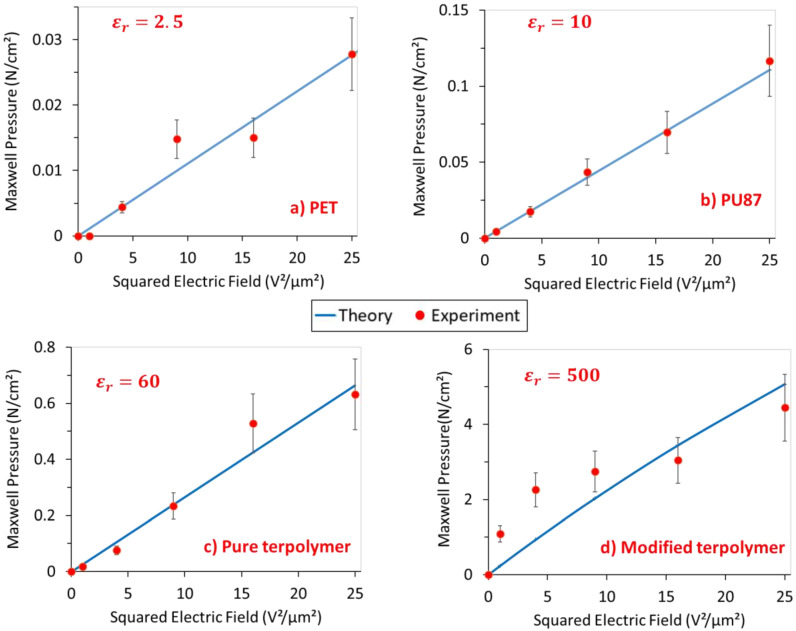
Experimental and theoretical data of Electroadhesion for different polymers comprising (**a**) PET; (**b**) PU87; (**c**) pure terpolymer; and (**d**) modified terpolymer.

In order to better assess the electroadhesion performances of all the four samples, measured data were gathered and compared to the expectations. Figure 8 displays experimental and theoretical Maxwell pressure (cf. Equation (3)) of (a) PET, (b) PU 87, (c) pure terpolymer, and (d) plasticized terpolymer. As expected, for all the four materials, empirical data are somehow similar to the theoretical model, confirming good reliability of the experimental setup. The results also allow to confirm the electroadhesion effect, which can be easily controlled through an application of the input voltage.

Considering these results, it can be concluded that for the PET (Figure 8a) and PU 87 (Figure 8b), the electroadhesion pressure is low, which is coherent to the estimations predicted on Section 2.4. It is also expected that their mechanical response is linear versus the squared electric field. As illustrated in Figure 8c, the pure terpolymer shows a similar trend, but with the Maxwell pressure 10 times higher compared to the case of the PET and PU 87. For all these three materials, good correlation between experiment and theory is achieved. The modified terpolymer (Figure 8d), however, displays performances that are slightly different from expected. Significant electrostatic forces are developed that could affect the material’s quality, leading to lower performances at higher fields. 

Discrepancies between theory and experiment are observed, which can be explained by several factors. The use of high voltage has a strong influence on the dielectric materials. The dielectric constant of electroactive polymers like PET, PU87, and pure terpolymer can somewhat decrease with the increasing electric field, even if not significantly [44], as opposed to the modified terpolymer. Furthermore, various phenomena like leaking current and Corona discharges in the dielectric materials can also cause a saturation of the performance under a high electric field [45]. Surface topography of the sample might also have an important impact on the results. Actually, the real surface of the electrode can be different from the nominal surface, making error in the pressure estimation. For instance, a smaller nominal area leads to smaller pressure given by dividing the measured force to an area that is overestimated. An explanation of this phenomenon has been reported in [46,47], which is dedicated to the mechanical contact’s theory of the electroadhesion. The same explanation can be provided for the roughness of the sample that is hardly controllable with the casting process, inducing uncertainty when measuring the sample’s thickness. This might provoke a slight error when calculating the electric field value, to some extent. In this study, for the sake of simplicity, only the normal component of the adhesion force has been considered. In reality, there might be a little tangential contribution that depends on the static friction. This limitation can explain discrepancies found between the empirical data and the theoretical model.

Accordingly, the measured Maxwell pressure of the modified terpolymer is 10 times higher than the pure one, and 100 times higher than both PU87 and PET. This confirms outstanding possibilities for the use of the modified terpolymer, especially in electroadhesion actuation.

Another alternative to validate the electroadhesion effect is relied on simulation investigation, that can be found on the supplementary materials (Title: Simulation of electroadhesion effect based COMSOL Multiphysics software).

**Figure 9:**
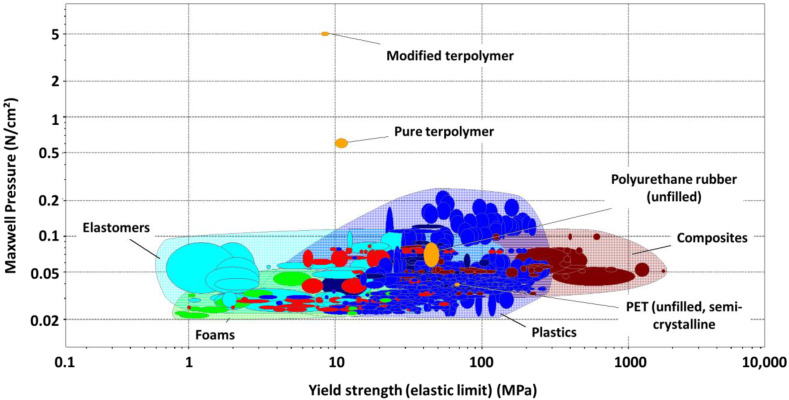
Maxwell pressure at 5 V/µm versus elastic limit for polymer and composite materials—studied materials are highlighted in orange.

Based on the Cambridge Engineering Selector (CES) EduPack 2020 library [48], it is possible to compare the adhesive actuation as well as the Yield strength (elastic limit) of our four developed materials among other classical polymeric and composites. Figure 9 illustrates the Maxwell pressure together with the elastic limit of all these materials. As predicted in Section 2.4, at a low electric field (i.e., 5 V/µm), the modified terpolymer leads to the best performance as a result of its excellent dielectric permittivity. However, it is noteworthy to pay attention to its relatively low elastic limit (i.e., around 8 MPa) as well as its saturation effect of the dipole’s polarization under high voltage application. The pure terpolymer exhibits somewhat higher elastic limit compared to the modified counterpart, but its Maxwell pressure is drastically lower, which is revealed by a factor of almost tenfold decrease under a 5 V/µm electric field. However, compared to other conventional polymers, the pure terpolymer seems to be an interesting alternative for application in electroadhesive actuation. Furthermore, this material has stable dielectric properties under a large range of the input voltage excitation. As a result, it can lead to a stable growth in the Maxwell pressure with respect to an increase in the electric field. Both PET and PU87 may be attractive solutions regarding their cost and high elastic limit. Nonetheless, they have a very weak electrostatic response that is coherent with their dielectric constant. Moreover, the breakdown strength of the PU87 is relatively low compared to the other materials (i.e., 50 V/µm), which limits its operating range of actuation and makes it not suitable for high voltage solicitation. Similar to the PET and PU87, elastomers and composites suffer from their poor electroadhesion effect. Significant efforts have been investigating among research communities to improve their performances [49,50,51]. However, these materials still require high electric field application to achieve satisfactory electrostatic force.

## 5. Applications to Active Control of Displacement and Stiffness for Electroactive Materials

One application of this phenomenon involves using the attractive Coulombian forces to create a linear movement of weights in a range of several micrometers to centimeters. As has already been investigated in some studies, this can be useful for a fine control of displacement in a micro [52] or macro-scale [53].Thanks to electroadhesion, it is possible to hold different weights regarding a certain electric field.

**Figure 10:**
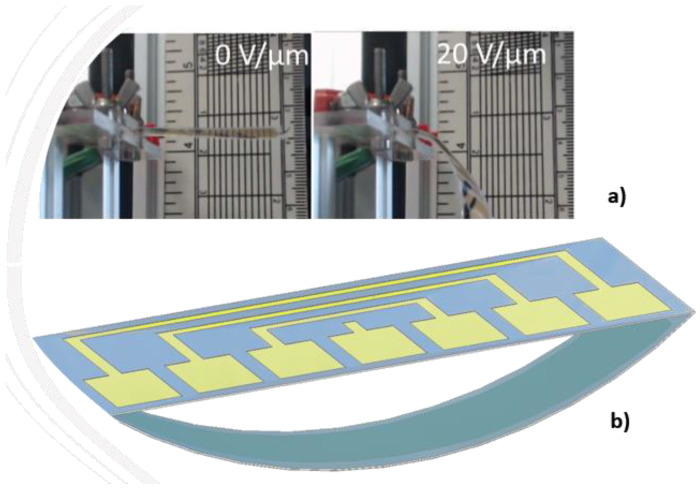
Active displacement control based on electrostrictive materials: (**a**) Actuator in flexion mode; (**b**) CAD design of an ideal benchmark.

Another application is relied on the active control of stiffness for electrostrictive materials. Under load, these materials exhibit a flexion that results in a tip displacement (Figure 10a). The system can be mechanically modelled as a spring under load. By varying the area of the active electrodes, it is possible to vary the range of the tip displacement and so does the stiffness of the equivalent spring. Figure 10b presents the CAD model of the ideal benchmark. Sectored electrodes and controllable electric power permit the variation of active electrode surfaces so as to achieve the active control of stiffness.

To demonstrate a feasibility of the device, experiments have been performed on two identical modified terpolymer that are clamped together on both sides. Variation of the electrode surface is made by manually removing one part of the electrodes from the below sample. As indicated in Equation (1), the smaller the active surface (denoted A as the electrode surface), the weaker the electroadhesive effect. Accordingly, changing the active surface leads to a variation of the resulting attractive force between two samples. In experiment, no weight has been fixed on the below sample. As a matter of fact, the electrostatic force may decrease due to the shrinking of the electrode surface, making it difficult to hold or move the weight. Table 2 described the experimental tests performed on the modified terpolymers, designed by three different patterns of the bottom electrode. Concretely,

- Table 2a displays a large rectangular-shaped electrode of (20×55) mm²;
- Table 2b dedicates to a symmetric structure comprising of two identical electrodes of (20×20) mm² situated on the two sides, while there is no active surface on the center;
- Table 2c is somehow similar to the design of Table 2b but with asymmetric structure, where the two electrodes have different dimensions of (5×20) mm² and (20×20) mm².
- Table 2d shows another symmetric structure similar to the one presented in Table 2b but with smaller electrodes surface of (5×5)mm².

**Table 2:** Evolution of displacement of the sample considering different patterns of electrodes.

Pattern of Bottom Electrode Sample with Dimension of (70 × 30) mm²	Picture	Tip Displacement
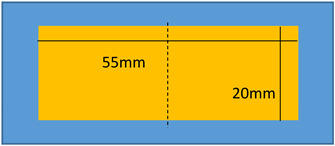 (a) Under an applied electric field, a generation of a static force on the whole active surface makes the two samples stick together. A tip displacement is recorded equal to 30 mm when the samples are subjected to an electric field of around 5 V/µm.	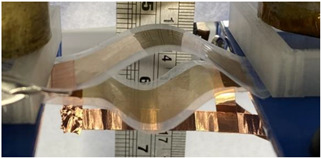	0 mm (0 V/µm)
	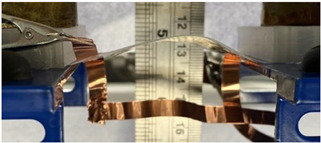	30 mm (~5 V/µm)
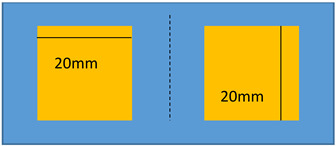 (b) Interestingly, when the input high voltage is ON, the two samples attract each other, but not on the whole surface. Obviously, the two side edges stick together, where there is a presence of the active electrodes. No actuation manifests at the center, which is logical based on the specified pattern design. A tip displacement of each side is equal to 15 mm, allowing to create a “sinus” form of the below sample.	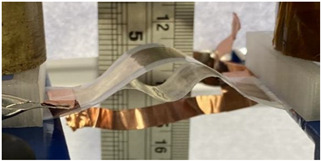	15 mm (~5 V/µm)
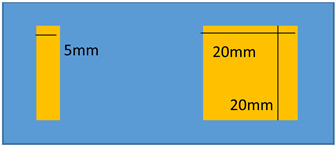 (c) When both samples are subjected to an electric field of approximately 5 V/µm, the two samples stick together only on the right-hand side, where the active surface is large enough to generate sufficient electrostatic force. The left-hand side, on the other hand, does not produce obvious electroadhesion phenomenon, especially when a part of the top electrode is removed from the above sample. Thus, the effective surface of the electrodes is too small to provoke any action. Interestingly, the resulting displacement of the below sample is assimilated to a “sinus” form, but with a period double to the one obtained from the case b.	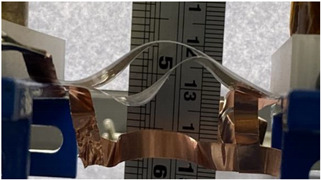	10 mm (~5 V/µm)
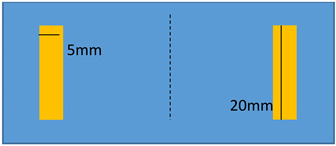 (d) When both samples are subjected to an electric field of approximately 5 V/µm, no displacement is observed. The effective surface of the electrodes is too small on both sides, and thus, no action can be provoked.	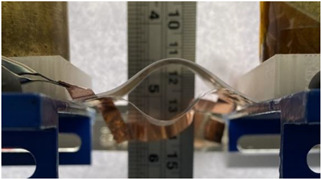	0 mm (~5 V/µm)

In each configuration, the displacement evolution of the below sample is observed when the applied electric field gradually increases from 0 to 5 V/µm, approximately. Its value is recorded when the below electrode is partially removed. Based on these observations, we can conclude that the higher the applied electric field, the higher the tip displacement and the stronger the attractive force. Interestingly, the effective areas are directly linked to the pattern of the electrodes surface. It is therefore possible to achieve a desired displacement of the tested sample by controlling the electrode’s design. For example, in a symmetric actuation (Table 2b), the tip displacement follows the sinus wave whose amplitude and frequency can be easily controlled. Actually, increasing the number of the sectored electrodes gives rise to increase frequency, while varying the input electric field leads to a change in the amplitude. In Table 2d, no action is observed as the surface of the two electrodes is too small.

In asymmetric actuation (Table 2c), border and buckling effects can be noticed on the below sample when the input electric filed suddenly passes from 0 to 5 V/µm. These effects are probably induced during the transient regime where the electromechanical response of the material somehow exhibits an overshoot, which is inevitable due to an abrupt variation of the voltage excitation. This behavior can be greatly subdued by gradually increasing the electric power or by adding the damping element to the system. In addition, on larger and heavier devices, the buckling issue might become negligible thanks to the weight effect.

## 6. Conclusions

This paper proposed a new criterion for material selection for electroadhesion performance, which is linked to a physical variable, the so-called Maxwell Pressure. Under a given electric field, this variable is revealed to be mainly affected by intrinsic parameters such as dielectric constant. However, the limitations of the breakdown electric field, the mechanical yield strength, as well as the surface roughness of the samples are also essential factors that have an impact on efficiency of the adhesive actuation. Another purpose of this work involved development of a reliable setup that enables to achieve accurate measurements of attractive forces. These forces are generated based on the opposite charges accumulated on the two samples’ surface, according to Coulomb’s law. Empirical data is in good correlation with the theoretical prediction, reflecting good reliability of the measurement method. Finally, the proposed approach is an efficient way to select polymer matrices with pertinent actuation properties, making it possible to simplify the comparison between several electroactive materials. The results in this study confirmed high potential of the developed material for real-world actuator applications, especially in multifunctional flexible electroadhesive devices.

Future work involves in development of a “Figure of Merit” for the normal electroadhesion by considering other relevant factors like the static friction, the roughness of the surface, and the dipole saturation effect. Further characterization of materials will be investigated, particularly focusing on evolution of electroadhesion performances under varying amplitude and frequency of Alternating Current (AC) solicitation.

## Figures and Tables

**Table 1:** Characteristics of different electroactive polymers.

Type	PET [41]	Pure Terpol. [13]	Modif. Terpol. [13]	PU 87 [42]
$\epsilon_{r}$ $\left(\propto \;P_{M}\right)$ $@1\;\mathrm{Hz}\;\mathrm{and}\;1\;V_{RMS}$	2.5	60	500	10
*µ*	0.3	1.01	0.7	6.5
Y (MPa)	400	100	45	20
$\mu \epsilon \;\left(\propto \;P_{F}\right)$	0.75	60.6	350	65
Breakdown field (V/µm)	80	270	140	50

## Data Availability

Not applicable.

## Supplementary Materials

The following are available online at https://zenodo.org/record/5643252 (accessed on 3 November 2021); Experimental setup including (**a**) Video S1: Holding a 200g weight using an applied field of 5.5 V/µm; and (**b**) Video S2: Lifting a 10g weight using an applied field of 15 V/µm; The following supporting information can be downloaded at: https://www.mdpi.com/article/10.3390/polym14010024/s1, Simulation of electroadhesion effect based COMSOL Multiphysics software. Figure S1: Simulation of Coulomb’s law experiment; Figure S2: (**a**) Mesh distribution and (**b**) Pressure distribution around the electrode; Figure S3: Electroadhesion effect of the pure terpolymer subjected to different levels of input electric field. (**a**) COMSOL simulation; (**b**) Comparison between experiment and simulation; Table S1: Characteristics of different electroactive polymers.

## References

[1] Johnsen A., Rahbek K. (1923). A Physical Phenomenon and Its Applications to Telegraphy, Telephony, Etc. J. Inst. Elect. Eng..

[2] Rahbek K. (1935). Electroadhesion Apparatus. U. S. Patent.

[3] Shintake J., Rosset S., Schubert B., Floreano D., Shea H. (2016). Versatile Soft Grippers with Intrinsic Electroadhesion Based on Multifunctional Polymer Actuators. Adv. Mater..

[4] Digumarti K.M., Cao C., Guo J., Conn A.T., Rossiter J. Multi-Directional Crawling Robot with Soft Actuators and Electroadhesive Grippers. Proceedings of the 2018 IEEE International Conference on Soft Robotics (RoboSoft).

[5] Shintake J., Cacucciolo V., Floreano D., Shea H. (2018). Soft Robotic Grippers. Adv. Mater..

[6] Graule M.A., Chirarattananon P., Fuller S.B., Jafferis N.T., Ma K.Y., Spenko M., Kornbluh R., Wood R.J. (2016). Perching and Takeoff of a Robotic Insect on Overhangs Using Switchable Electrostatic Adhesion. Science.

[7] Guo J., Xiang C., Rossiter J. (2018). A Soft and Shape-Adaptive Electroadhesive Composite Gripper with Proprioceptive and Exteroceptive Capabilities. Mater. Des..

[8] Heath C.J.C., Bond I.P., Potter K.D., Farinholt K.M., Griffin S.F. (2015). Integrating Electrostatic Adhesion to Composite Structures. Industrial and Commercial Applications of Smart Structures Technologies 2015, Proceedings of SPIE Smart Structures and Materials + Nondestructive Evaluation and Health Monitoring, San Diego, CA, USA, 1 April 2015.

[9] Han A.K., Hajj-Ahmad A., Cutkosky M.R. (2021). Hybrid Electrostatic and Gecko-Inspired Gripping Pads for Manipulating Bulky, Non-Smooth Items. Smart Mater. Struct..

[10] Le M.Q., Ganet F., Audigier D., Capsal J.-F., Cottinet P.-J. (2017). Printing of Microstructure Strain Sensor for Structural Health Monitoring. Appl. Phys. A.

[11] Thetpraphi K., Kanlayakan W., Chaipo S., Moretto G., Kuhn J., Audigier D., Le M.Q., Cottinet P.-J., Petit L., Capsal J.-F. (2021). 3D-Printed Electroactive Polymer Force-Actuator for Large and High Precise Optical Mirror Applications. Addit. Manuf..

[12] Bauer S., Bauer-Gogonea S., Graz I., Kaltenbrunner M., Keplinger C., Schwödiauer R. (2014). 25th Anniversary Article: A Soft Future: From Robots and Sensor Skin to Energy Harvesters. Adv. Mater..

[13] Rus D., Tolley M.T. (2015). Design, Fabrication and Control of Soft Robots. Nature.

[14] Bar-Cohen Y., Anderson I.A. (2019). Electroactive Polymer (EAP) Actuators—Background Review. Mech. Soft Mater..

[15] Capsal J.-F., Galineau J., Lallart M., Cottinet P.-J., Guyomar D. (2014). Plasticized Relaxor Ferroelectric Terpolymer: Toward Giant Electrostriction, High Mechanical Energy and Low Electric Field Actuators. Sens. Actuators A Phys..

[16] Bar-cohen Y. Electroactive Polymers as Artificial Muscles—Reality and Challenges. Proceedings of the 19th AIAA Applied Aerodynamics Conference.

[17] Thetpraphi K., Le M.Q., Houachtia A., Cottinet P., Petit L., Audigier D., Kuhn J., Moretto G., Capsal J. (2019). Surface Correction Control Based on Plasticized Multilayer P(VDF-TrFE-CFE) Actuator—Live Mirror. Adv. Opt. Mater..

[18] Schiava N.D., Pedroli F., Thetpraphi K., Flocchini A., Le M.-Q., Lermusiaux P., Capsal J.-F., Cottinet P.-J. (2020). Effect of Beta-Based Sterilization on P(VDF-TrFE-CFE) Terpolymer for Medical Applications. Sci. Rep..

[19] Wang T., Farajollahi M., Choi Y.S., Lin I.-T., Marshall J.E., Thompson N.M., Kar-Narayan S., Madden J.D.W., Smoukov S.K. (2016). Electroactive Polymers for Sensing. Interface Focus.

[20] Jung K., Kim K.J., Choi H.R. (2008). A Self-Sensing Dielectric Elastomer Actuator. Sens. Actuators A Phys..

[21] Ganet F., Le M.Q., Capsal J.F., Lermusiaux P., Petit L., Millon A., Cottinet P.J. (2016). Development of a Smart Guide Wire Using an Electrostrictive Polymer: Option for Steerable Orientation and Force Feedback. Sci. Rep..

[22] Liu Q., Le M.Q., Richard C., Liang R., Cottinet P.-J., Capsal J.-F. (2019). Enhanced Pseudo-Piezoelectric Dynamic Force Sensors Based on Inkjet-Printed Electrostrictive Terpolymer. Org. Electron..

[23] Cottinet P.-J., Le M.-Q., Degraff J., Souders C., Liang Z., Wang B., Zhang C. (2013). Strain Phenomenon in Carbon Nanotube Buckpaper Actuator: Experiments and Modeling. Sens. Actuators A Phys..

[24] Mokni M., Pedroli F., D’Ambrogio G., Le M.-Q., Cottinet P.-J., Capsal J.-F. (2020). High-Capacity, Fast-Response, and Photocapacitor-Based Terpolymer Phosphor Composite. Polymers.

[25] Hinchet R., Shea H. (2020). High Force Density Textile Electrostatic Clutch. Adv. Mater. Technol..

[26] Capsal J.-F., Galineau J., Le M.-Q., Dos Santos F.D., Cottinet P.-J. (2015). Enhanced Electrostriction Based on Plasticized Relaxor Ferroelectric P(VDF-TrFE-CFE/CTFE) Blends. J. Polym. Sci. Part B Polym. Phys..

[27] Strong R., Troxel D. (1970). An Electrotactile Display. IEEE Trans. Man Mach. Syst..

[28] Ivkovic B., Djurdjanovic M., Stamenkovic D. (2000). The Influence of the Contact Surface Roughness on the Static Friction Coefficient. Tribol. Ind..

[29] Lessel M., Loskill P., Hausen F., Gosvami N.N., Bennewitz R., Jacobs K. (2013). Impact of van Der Waals Interactions on Single Asperity Friction. Phys. Rev. Lett..

[30] Rubinstein S.M., Cohen G., Fineberg J. (2006). Contact Area Measurements Reveal Loading-History Dependence of Static Friction. Phys. Rev. Lett..

[31] Parker R.C., Hatch D. (1950). The Static Coefficient of Friction and the Area of Contact. Proc. Phys. Soc. Sect. B.

[32] Bowden F.P., Tabor D. (2001). The Friction and Lubrication of Solid.

[33] Hulikal S., Lapusta N., Bhattacharya K. (2018). Static and Sliding Contact of Rough Surfaces: Effect of Asperity-Scale Properties and Long-Range Elastic Interactions. J. Mech. Phys. Solids.

[34] Pedroli F., Flocchini A., Marrani A., Le M.-Q., Sanseau O., Cottinet P.-J., Capsal J.-F. (2020). Boosted Energy-Storage Efficiency by Controlling Conduction Loss of Multilayered Polymeric Capacitors. Mater. Des..

[35] Pedroli F., Marrani A., Le M.-Q., Sanseau O., Cottinet P.-J., Capsal J.-F. (2019). Reducing Leakage Current and Dielectric Losses of Electroactive Polymers through Electro-Annealing for High-Voltage Actuation. RSC Adv..

[36] Della Schiava N., Le M.-Q., Galineau J., Dos Santos F.D., Cottinet P.-J., Capsal J.-F. (2017). Influence of Plasticizers on the Electromechanical Behavior of a P(VDF-TrFE-CTFE) Terpolymer: Toward a High Performance of Electrostrictive Blends. J. Polym. Sci. Part B Polym. Phys..

[37] Schiava N.D., Thetpraphi K., Le M.-Q., Lermusiaux P., Millon A., Capsal J.-F., Cottinet P.-J. (2018). Enhanced Figures of Merit for a High-Performing Actuator in Electrostrictive Materials. Polymers.

[38] Le M.Q., Capsal J.-F., Galineau J., Ganet F., Yin X., Yang M., Chateaux J.-F., Renaud L., Malhaire C., Cottinet P.-J. (2015). All-Organic Electrostrictive Polymer Composites with Low Driving Electrical Voltages for Micro-Fluidic Pump Applications. Sci. Rep..

[39] Ganet F., Le M.-Q., Capsal J.F., Gérard J.F., Pruvost S., Duchet J., Livi S., Lermusiaux P., Millon A., Cottinet P.-J. (2015). Haptic Feedback Using an All-Organic Electroactive Polymer Composite. Sens. Actuators B Chem..

[40] Meijer H.E.H., Govaert L.E. (2005). Mechanical Performance of Polymer Systems: The Relation between Structure and Properties. Prog. Polym. Sci..

[41] Grzybowski S., Feilat E.A., Knight P., Doriott L. Breakdown Voltage Behavior of PET Thermoplastics at DC and AC Voltages. Proceedings of the Proceedings IEEE Southeastcon’99. Technology on the Brink of 2000 (Cat. No.99CH36300).

[42] Karlsson M. (2014). Investigation of the Dielectric Breakdown Strength of Polymer Nanocomposites. Master’s Thesis.

[43] Capsal J.-F., Lallart M., Galineau J., Cottinet P.-J., Sebald G., Guyomar D. (2012). Evaluation of Macroscopic Polarization and Actuation Abilities of Electrostrictive Dipolar Polymers Using the Microscopic Debye/Langevin Formalism. J. Phys. D Appl. Phys..

[44] Furukawa T., Nakajima K., Koizumi T., Date M. (1987). Measurements of Nonlinear Dielectricity in Ferroelectric Polymers. Jpn. J. Appl. Phys..

[45] Koh K., Sreekumar M., Ponnambalam S. (2014). Experimental Investigation of the Effect of the Driving Voltage of an Electroadhesion Actuator. Materials.

[46] Ciavarella M., Papangelo A. (2020). A Simplified Theory of Electroadhesion for Rough Interfaces. Front. Mech. Eng..

[47] Persson B.N.J. (2018). The Dependency of Adhesion and Friction on Electrostatic Attraction. J. Chem. Phys..

[48] GRANTA Edupack. https://grantadesign.com/education/ces-edupack/.

[49] Lau G.-K., Heng K.-R., Ahmed A.S., Shrestha M. (2017). Dielectric Elastomer Fingers for Versatile Grasping and Nimble Pinching. Appl. Phys. Lett..

[50] Lim H., Hwang G., Kyung K.-U., Kim B.-J. (2021). Improved Electroadhesive Force by Using Fumed Alumina/PDMS Composites. Smart Mater. Struct..

[51] Heath C.J.C., Bond I.P., Potter K.D. (2016). Electrostatic Adhesion for Added Functionality of Composite Structures. Smart Mater. Struct..

[52] Maffli L., Rosset S., Shea H.R. (2013). Zipping Dielectric Elastomer Actuators: Characterization, Design and Modeling. Smart Mater. Struct..

[53] Taghavi M., Helps T., Rossiter J. (2018). Electro-Ribbon Actuators and Electro-Origami Robots. Sci. Robot..

