# Supplementary material for: Accurate Electroadhesion Force Measurements of Electrostrictive Polymers: The Case of High Performance Plasticized Terpolymers
Source: Polymers (Basel). 2021 Dec 22;14(1):24. doi: 10.3390/polym14010024 (PMC8747691; doi:10.3390/polym14010024)
Supplement: Supplementary file 1 [file polymers-14-00024-s001.zip › polymers-1471059-supplementary.pdf]

Accurate Electroadhesion Force Measurements of Electrostrictive Polymers: The Case of High Performance Plasticized Terpolymers

Supporting Information

Simulation of electroadhesion effect based COMSOL Multiphysics software

Another alternative to validate the electroadhesion effect is relied on simulation investigation. In the electromechanics module of the software COMSOL Multiphysics (abbreviated as COMSOL), two samples have been built with the same dimension of 100µm thickness, 50 mm length, and 30 mm width. A circular electrode of 20mm diameter is coated on the top of the above sample while the bottom surface of the below sample is assimilated to the ground electrode as depicted in *Figure S1*. These samples are set as "Linear Elastic Dielectric" material in Comsol interface and a Zero charge condition is set for all the boundaries, except the ones of the electrodes.

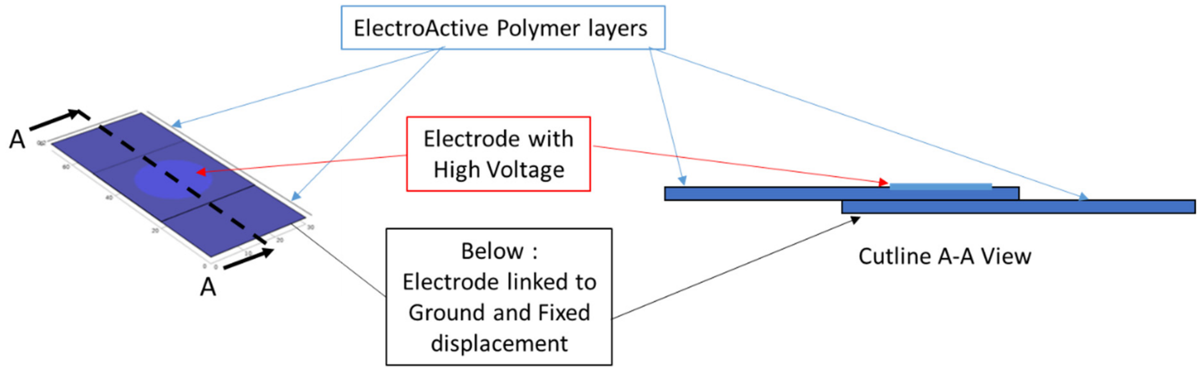

Figure S1. Simulation of Coulomb's law experiment.

Different levels of direct current (DC) voltage are applied between the two electrodes, corresponding to electric fields in the range of [0-20] V/µm. The chosen magnitude of the electric field is quite low because within this range, the elastic and dielectric properties of the model is considered to be linear. Coulomb's law is then calculated automatically and integrated inside simulation creating Maxwell Pressure. To simulate the weights exerting on the below sample, a fixed constraint is subjected to its bottom surface.

The size of triangular mesh is automatically generated by finite element method, fine enough to obtain accurate calculation of the pressure induced from electrostatic force (see Figure S2a). It is worth noting that the force distribution is not precise around the electrode area because of the chosen mesh, whose border effects are negligible in this approach. Figure S2b gives an overview of the pressure distribution on the electrode and its surrounding area, confirming that actuation only occurs between the electrodes surface.

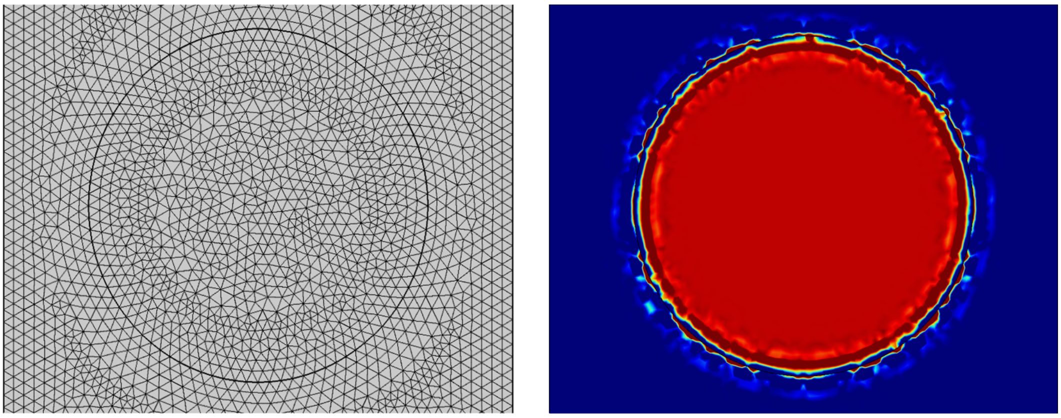

(a) (b)

Figure S2. (**a**) Mesh distribution and (**b**) Pressure distribution around the electrode

Simulation is computed in different model of materials (PET, PU87, pure and modified terpolymers) whose characteristics are given in Table S1. However, for a sake of simplicity, only results performed with the pure terpolymer is presented in this analysis. A full comparison of all materials, comprising both empirical data and COMSOL simulation, is thoroughly carried out in the main article.

Table S1. Characteristics of different electroactive polymers.

TYPE	PET [38]	PURE TERPOL. [13]	MODIF. TERPOL. [13]	PU 87 [39]
ϵ_r @1HZ AND 1 V_{RMS}	2.5	60	500	10
µ	0.3	1.01	0.7	6.5
Y (MPA)	400	100	45	20
$\mu\epsilon$ $(i.e. FoM_{Stress})$	0.75	60.6	350	65
BREAKDOWN FIELD (V/µM)	80	150	140	50

The results can be found on Figure S3a, showing the surface pressure at different levels of the electric field. Figure S3b displays experimental and simulated behavior of the Maxwell pressure as a function of the squared electric field. The result highlights good linear dependence between them, which agrees with the typical electrostrictive properties of the terpolymer. Experimental setup seems to provide accurate results concerning the evaluation of Coulomb's electrostatic forces and therefore Maxwell Pressure.

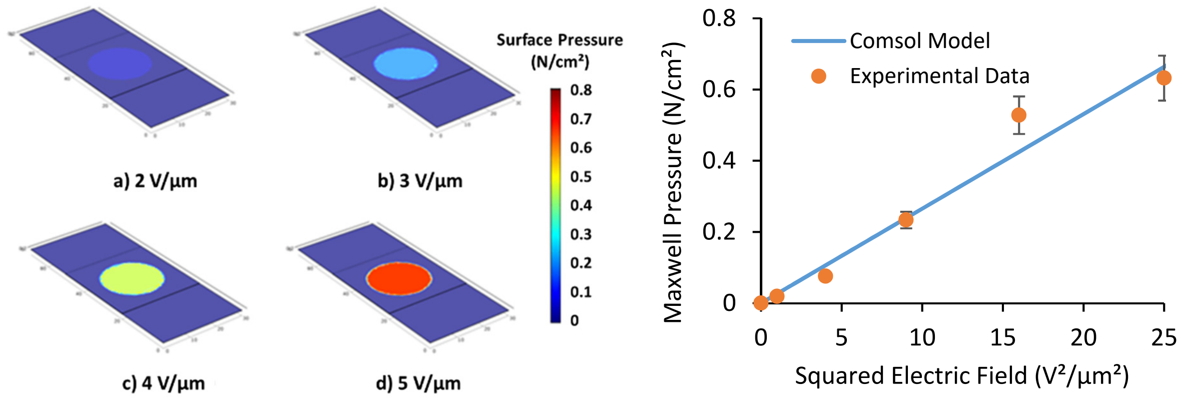

(a) (b)

Figure S3 : Electroadhesion effect of the pure terpolymer subjected to different levels of input electric field. (**a**) COMSOL simulation; (**b**) Comparison between experiment and simulation.
